# Posttranscriptional Regulation of the Plasminogen Activation System by Non-Coding RNA in Cancer

**DOI:** 10.3390/ijms24020962

**Published:** 2023-01-04

**Authors:** Mariaevelina Alfieri, Luigia Meo, Pia Ragno

**Affiliations:** 1 Clinical Pathology, Pausilipon Hospital, A.O.R.N Santobono-Pausilipon, 80123 Naples, Italy; 2 Department of Chemistry and Biology, University of Salerno, Via Giovanni Paolo II, 132, 84084 Fisciano, Italy

**Keywords:** plasminogen activation system, uPAR, uPA, PAI-1, PAI-2, non-coding RNA, microRNA, ceRNA

## Abstract

Various species of non-coding RNAs (ncRNAs) may act as functional molecules regulating diverse biological processes. In cancer cell biology, ncRNAs include RNAs that regulate the expression of oncogenes and tumor suppressor genes through various mechanisms. The urokinase (uPA)-mediated plasminogen activation system (PAS) includes uPA, its inhibitors PAI-1 and PAI-2 and its specific cellular receptor uPAR; their increased expression represents a negative prognostic factor in several cancers. Here, we will briefly describe the main uPA-mediated PAS components and ncRNA species; then, we will review more recent evidence of the roles that ncRNAs may play in regulating the expression and functions of uPA-mediated PAS components in cancer.

## 1. Introduction

In the nineteenth century, medical scientists could only observe, weigh and measure tumors; however, despite the lack of useful tools, Rudolf Virchow deduced the cellular origin of cancer (1863) and Stephen Paget proposed the seed-and-soil hypothesis of metastasis (1889). Only in the following century, did Peyton Rous propose the viral origin of avian cancer (1911) and Theodor Boveri hypothesize that cancer might be associated with chromosomal mutations [1]. Many key advances followed these crucial intuitions, allowed by the extraordinary progress made in biochemistry, genetics and cell and molecular biology. For a long time, the central dogma of biology was that genetic information was transmitted from DNA to RNA, leading to protein synthesis. This dogma guided cancer research, which focused mainly on protein-coding genes. However, only less than 2% of the human genome encodes proteins, even though more than 40% of the genome is in fact transcribed. This evidence indicates that the larger part of the human transcriptome consists of non-coding RNAs (ncRNAs) [2]. Indeed, transfer RNA (tRNA) and ribosomal RNA (rRNA), which are not translated into proteins, have been well characterized in their structure and function in the past, but only in the last two decades, a plethora of studies has focused on the structural and functional characterization of new forms of ncRNAs [3]. In the context of cancer cell biology, ncRNAs include RNAs regulating the expression of oncogenes and tumor suppressor genes through various mechanisms, thus acting themselves as tumor suppressors or oncogenes [4]. 

The urokinase (uPA)-mediated plasminogen activation system (PAS) comprises various components, including two plasminogen activators, two inhibitors, the specific uPA receptor (uPAR) and, of course, plasminogen, a circulating zymogen which can be converted to plasmin, a broad-spectrum serine protease [5]. For a long time, the uPA-mediated PAS was associated with cancer invasion and metastasis exclusively for its ability to promote the focused degradation of the extracellular matrix (ECM), allowing tumor cells to infiltrate the surrounding tissues and to disseminate in the organism. Interestingly, a large body of evidence has now clearly demonstrated that some components of the uPA-mediated PAS are also able to sustain tumor progression independently of proteolysis [6].

Here, we will briefly describe the main uPA-mediated PAS components at the protein level and the main ncRNA species; then, we will review the roles that ncRNAs may play in regulating the expression and functions of uPA-mediated PAS components in cancer.

## 2. The Plasminogen Activation System

### 2.1. Plasminogen/Plasmin

Plasminogen is synthesized in the liver as a single chain glycoprotein and then released into the bloodstream. It is converted to plasmin only when its proteolytic activity is required. Activation consists of a single cleavage at Arg561–Val562, which generates a two-chain molecule; the N-terminal heavy chain is characterized by five kringle domains while the C-terminal light chain contains the catalytic site. Plasminogen/plasmin kringle domains contain lysine binding sites that mediate plasminogen/plasmin binding to fibrin, to the physiological inhibitor alpha 2-antiplasmin or to the cell surface [5,7].

Plasmin is a potent fibrinolytic agent. Plasmin also promotes ECM degradation, both directly, by degrading ECM components, and indirectly, by activating MMPs. Furthermore, it can promote the cleavage/activation or release of cell adhesion molecules and/or growth factors, thus indirectly regulating cell adhesion and migration and cell proliferation [5,8].

Plasminogen activation needs to be tightly regulated because of these broad and important plasmin activities. In fact, plasminogen is physiologically activated by two specific plasminogen activators, the tissue-type (tPA) and the urokinase type (uPA).

### 2.2. Plasminogen Activators

Both uPA and tPA are secreted as single chains which, by a single cleavage, generate two-chain enzymes. Single-chain tPA exerts a significant activity that increases after cleavage. By contrast, the single-chain pro-uPA is inactive and can activate plasminogen only after cleavage at the Lys^158^-Ile^159^ residues. This cleavage generates the A chain, whose amino-terminal fragment (ATF, amino acids 1–135) binds a specific cellular receptor (uPAR), and the B chain, containing the catalytic site that is able to cleave and to activate plasminogen [7].

Single-chain tPA can bind fibrin, which also binds plasminogen. The formation of a ternary complex containing enzymes and substrates strongly enhances fibrinolysis. Thus, tPA has long been considered crucial in fibrinolysis and it is probably still viewed by most as such. However, over the years, tPA has been shown to play a role in several other physiological and pathological processes [9].

Single-chain uPA does not bind fibrin, although it probably cooperates with tPA in fibrinolysis [10]. However, the identification of a highly specific cellular receptor for uPA (uPAR) suggested a crucial role for this plasminogen activator in the non-fibrinolytic activities of plasmin, in particular in ECM degradation, a central activity in cell migration [11]. Over the years, other important roles have been demonstrated for uPA, independently of its proteolytic activity. In fact, inactivated uPA or its ATF can activate intracellular signaling pathways leading to cell migration, increased cell adhesion, proliferation and survival [12]. Consistently, increased levels in uPA are observed in several cancer types and represent a poor prognostic factor [13].

### 2.3. Inhibitors

PAS activity must be highly regulated because of its strong biological effects. The main inhibitor of free plasmin is the α2-antiplasmin [7].

Both uPA and tPA activity can be regulated by two specific inhibitors, type 1 (PAI-1) and type 2 (PAI-2) plasminogen activator inhibitors. 

PAI-1 is the main serpin (serine protease inhibitor) inhibiting uPA; it also binds two different domains of vitronectin (VN), an ECM component [14,15].

PAI-1 inhibits uPA, thus impairing plasminogen activation and, consequently, ECM degradation. However, contrary to what would be expected, PAI-1 expression increases in various cancers and its increase is associated with poor prognosis. This paradox could be partly due to the ability of PAI-1 to limit uPA proteolytic activity, thus preserving the three-dimensional ECM scaffold necessary for cell migration. PAI-1 is also involved in cell migration through its ability to regulate dynamic cell adhesion to the ECM during cell migration. In fact, PAI-1 can bind the low-density lipoprotein receptor-related protein 1 (LRP1), which is an important endocytic receptor for multiple ligands. In particular, LRP-1 associates with PAI-1 complexed to uPAR-bound uPA, thus mediating the endocytosis of the PAI-1–uPA–uPAR complex. uPA and PAI-1 are then degraded in lysosomes, whereas uPAR is recycled to the cell surface, ready to function again. PAI-1 also contributes to cancer progression by promoting angiogenesis through similar mechanisms [16,17].

PAI-2 has been detected in two different forms, a secreted 60 kDa glycosylated form and a 47 kDa non-glycosylated intracellular form. The role of PAI-2 in cancer is controversial. In fact, high levels of PAI-2 in various cancer types are associated with reduced cancer progression and metastasis [18]. Consistently, PAI-2 deficiency promotes spontaneous tumorigenesis and growth of melanoma and Lewis lung carcinoma cells in mouse models [19]. However, high levels of PAI-2 were also associated with increased lymph node metastasis and lower survival in breast cancer patients [20]. Furthermore, PAI-2 supported bladder cancer progression in PAI-1 knockout mice, suggesting functional redundancy [21].

PA inhibitors impair the activity of uPA, both in its free form and when bound to the cell surface through its specific receptor, uPAR, which, over the years, has progressively attracted attention for its several and diverse activities, playing a central role in the PA system.

### 2.4. The uPA Receptor 

The uPAR protein was identified in monocytes in 1985 [11] and its cDNA was sequenced five years later [22]. The mature uPAR protein is a 283-amino acid polypeptide chain organized in a three-domain structure, carrying a glycosylphosphatidylinositol (GPI) tail. The GPI tail anchors the receptor to the cell membrane and contributes in regulating its functions [23]. uPAR concentrates uPA on the cell surface, where low-affinity plasminogen binding sites concentrate the uPA substrate, leading to enhanced pericellular proteolysis [24]. However, proteolysis-independent uPAR activities have been clearly demonstrated. In fact, uPAR also acts as an adhesion receptor for VN. uPAR-bound uPA and VN are able to activate intracellular signaling pathways regulating cell adhesion, migration, proliferation and survival [25]. In fact, uPAR also interacts with various cell surface molecules, regulating their activity, in particular with integrins and with the chemotaxis receptors for formylated peptides (FPRs). Interactions with integrins and FPRs allow uPAR to signal inside the cell, despite its GPI tail. The multiple uPAR activities can potentially contribute to all cancer hallmarks. Consistently, uPAR expression increases in several cancers; this increase is associated with a more aggressive cancer behaviour and, as with uPA and PAI-1, it represents a negative prognostic factor [26].

**Figure 1:**
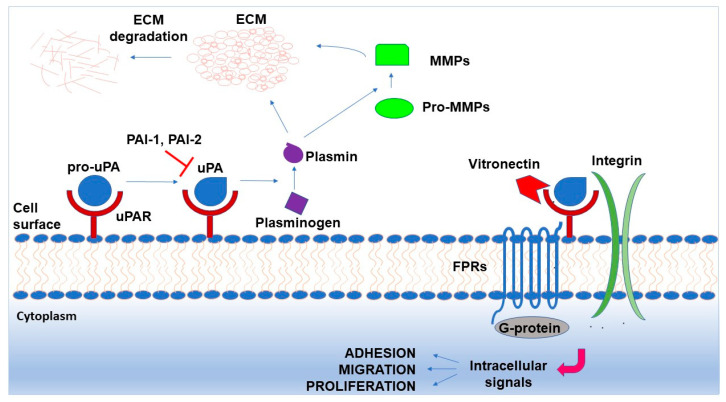
Plasminogen activation system: components, interactions and functions. Urokinase (uPA) is secreted as pro-enzyme (pro-uPA), binds its receptor (uPAR) and is activated. Active uPA is inhibited by two specific inhibitors, type 1 and type 2 (PAI-1 and PAI-2, respectively). The uPA enzyme converts plasminogen to plasmin that, in turn, promotes the degradation of extracellular matrix (ECM) components, directly or by activating matrix metalloproteases (MMPs). The uPAR protein also binds extracellular vitronectin. Moreover, uPAR can activate intracellular signaling pathways through interactions with integrins and G-coupled receptors for formylated peptides (FPRs). Activated signals regulate cell adhesion, migration and proliferation.

In conclusion, most of the components of the PA system, represented in Figure 1, are involved in cancer biology and their expression is upregulated in various cancers. This increase can occur at the transcriptional and posttranscriptional levels. In this review, we will focus on the role of ncRNAs in the posttranscriptional regulation of the components of the uPA-mediated PAS.

## 3. ncRNAs in Posttranscriptional Regulation

In general, ncRNAs play critical roles in multiple regulatory processes, including transcription, posttranscriptional modifications and translation. Based on their size, ncRNAs can be divided into two main classes: the small ncRNAs, including microRNAs (miRNAs), tRNA-derived small RNAs (tsRNAs), piwi-interacting RNAs (piRNAs), and the long ncRNAs (lncRNAs), untranslated RNAs greater than 200 nt in length, including pseudogenes and circular RNAs (circRNAs), which are single-stranded covalently closed RNAs [4]. 

MicroRNAs are the most extensively studied small ncRNAs. MicroRNAs are 22 nt ncRNAs that regulate gene expression by binding complementary sequences in target mRNAs. They are transcribed as pri-miRNAs, which are then cleaved into 60 nt pre-miRNAs by a complex containing Drosha and DGCR8, before being exported to the cytoplasm, where they are cleaved by Dicer to form miRNA duplexes. The guide strand of the miRNA duplex is loaded onto an Argonaute protein associated to an RNA-induced silencing complex (RISC). Mature miRNAs bind specific sequences in the 3′ untranslated regions (3′UTR) of target mRNAs, thereby impairing their translation and/or inducing their degradation [27]. 

The expression of more than 60% of human protein-coding genes is predicted to be regulated by miRNAs [28]. miRNAs can act on various and different mRNAs; at the same time, their targets can bind various and different miRNAs. A consequence of this complex network is that an mRNA targeted by specific miRNAs can compete with other mRNAs targeted by the same miRNAs, thus regulating their availability in the cytoplasm. This mRNA, indicated as a “competitive endogenous RNA” (ceRNA), can recruit specific miRNAs, thereby liberating other target mRNAs, which are allowed to be translated. Indeed, besides protein-coding mRNAs, other RNA species can act as ceRNAs, including lncRNAs, circRNAs and transcripts of pseudogenes [29].

A further layer of complexity is due to the fact that miRs generally bind specific sequences located in the 3′UTR of target mRNAs. The 3′UTR can also be targeted by other posttranscriptional regulators of gene expression, such as RNA binding proteins (RBPs). RBPs are able to regulate the stability of targeted mRNAs, thus promoting or impairing their degradation [30]. RBP-mediated posttranscriptional regulation may thus overlap, contribute to or interfere with miR-mediated posttranscriptional regulation. 

lncRNAs include heterogeneous intergenic transcripts, enhancer RNAs (eRNAs) and sense or antisense transcripts that overlap with other genes. Recently, it has been shown that some transcripts known as lncRNAs encode small proteins. Moreover, lncRNAs may influence gene expression at the transcriptional and posttranscriptional levels. Some lncRNAs act as a flexible molecular scaffold that brings together regulatory molecules such as RNAs, DNA and proteins, allowing for their interactions and biological activities. For instance, one of the reported roles of lncRNAs is in driving chromatin-modifying complexes to target gene promoters to influence transcriptional repression/activation; another role is to mediate the binding of RBPs that regulate mRNA processing and stability to their target mRNAs. Indeed, lncRNAs can also function as molecular sponges, binding mRNAs or miRNAs to modulate their intracellular levels or to impair mRNA translation [31].

circRNAs are a particular subgroup of lncRNAs. They are often generated from intronic or exonic sequences through a back-splicing process. Their functions and the mechanisms regulating their activity are still not fully elucidated, although they appear to be similar to those of lncRNAs. In particular, circRNAs can recruit miRNAs, thus acting as molecular sponges, or they can bind proteins directly, acting as a scaffold for molecular assembly [32].

**Figure 2:**
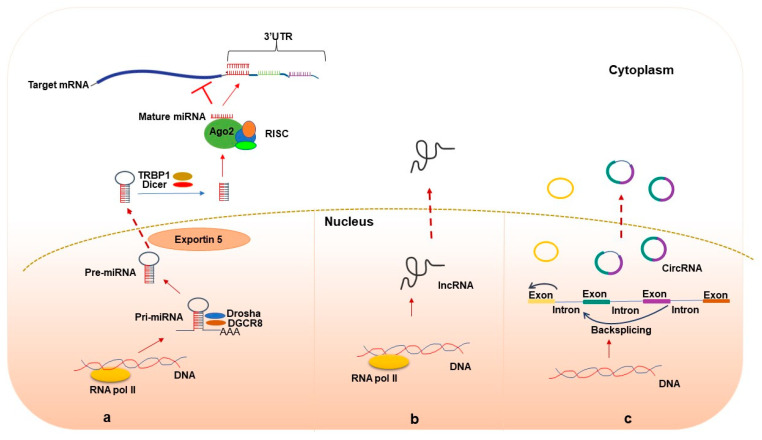
Biogenesis of the main non-coding RNA species. (**a**) miRNAs are transcribed in the nucleus by RNA polymerase II (RNA pol II) as pri-miRNAs. Pri-miRNAs are cleaved by a complex containing Drosha and DGCR8, generating pre-miRNA. The pre-miRNA is exported to the cytoplasm by Exportin 5, where it is cleaved by Dicer, to form a miRNA duplex. The guide strand of the miRNA duplex is loaded onto an Argonaute protein in the RNA-induced silencing complex (RISC). The mature miRNA binds specific sequences in the 3′ untranslated regions (3′UTR) of target mRNAs. (**b**) Long non-coding RNAs (lncRNAs) are heterogeneous RNAs transcribed by RNA pol II from independent promoters. (**c**) circRNAs are lncRNAs often generated from intronic or exonic sequences through a back-splicing process.

The biogenesis of the main ncRNAs is represented in Figure 2.

The intricate network connecting different RNA species results in a potent and flexible mechanism for regulating gene expression. In fact, small and long ncRNAs are key regulators of gene expression in many different cellular pathways and systems and have been implicated in several diseases, including cancer. 

### ncRNAs in Cancer

Dysregulation of the expression of both small and long ncRNAs has been observed in several malignancies, where they can play an oncogenic or tumor suppressor role, depending on which process or molecule is targeted, influencing multiple aspects of cancer biology such as tumor cell growth, cell death resistance, metabolism, invasion and metastasis. Interestingly, in some cases, the same ncRNA can function as either a tumor suppressor or oncogene, according to the context in which it is working [4]. 

Various miRNAS are differentially expressed in the tumoral tissues and bodily fluids of cancer patients, compared to normal tissues or healthy controls. Circulating miRNAs can be loaded into extracellular vesicles (EVs), where they are protected from RNases. The profiles of miRNA expression in serum EVs and in serum can be different and both are probably important as cancer biomarkers. Numerous miRs are potential predictive markers of drug response, response to immunotherapy, response to radiotherapy or represent diagnostic/prognostic markers in various cancers including lung, breast, prostate, colorectal, oral carcinomas, lymphomas and leukemias [33].

The levels of several circRNAs are also associated with tumor stage or differentiation in gastric, colorectal, hepatocellular and bladder carcinomas and have been proposed as biomarkers for the diagnosis, prognosis and monitoring of treatment responses [34].

Other tissue-specific lncRNAs differentially expressed in some cancers represent potential biomarkers for cancer diagnosis or prognosis. MALAT1 and HOTAIR have been approved as diagnostic markers in gastric carcinoma and PCA3 as a biomarker for the early diagnosis and prognosis of prostate cancer [35,36]. 

Their ability to regulate the expression of multiple target genes also makes ncRNAs promising targets/tools in cancer therapy. Among the ncRNAs, miRs are the most studied. Strategies of miR-based therapy include the inhibition of oncogenic miRs and the use of oncosuppressor miR mimics. 

miR inhibitors are oligonucleotides complementary to the specific miR (anti-miR oligonucleotides: AMOs). AMOs impair miR interaction with the target mRNA and promote its degradation. AMOs can be chemically modified in locked nucleic acid (LNA) to assume a conformation improving their stability and efficiency. Another strategy to inhibit the action of oncogenic miRs is the miR sponge. The miR sponge is a RNA containing multiple complementary binding sites for a target miR and is thus able to recruit it and inhibit its activity.

On the other hand, miR mimics are small double-stranded RNA molecules that mimic endogenous oncosuppressor miRNAs, designed to restore the lost activity of downregulated oncosuppressor miRs. 

Various miRNA-based anti-cancer strategies appear to be promising and many studies are in progress to identify the most suitable delivery system for miR mimics and miR inhibitors. However, the main issue still remains in the off-target effects of miR-based therapeutics, which need to be addressed in order to reduce their toxicity without affecting their therapeutic effects. To date, only 10 miR-based molecules have been tested in clinical trials and none of them has entered into phase III clinical trials [37].

## 4. ncRNAs in the Plasminogen Activation System

### 4.1. uPA

uPA is expressed in different tissues at low levels; its expression can be upregulated by growth factors, cytokines, hormones and cell morphology changes, both at the transcriptional and posttranscriptional levels. Increased uPA is observed in various cancers and represents a negative prognostic factor [12]. 

uPA overexpression is associated with poor prognosis in gastric and colorectal cancer. Gastric adenocarcinoma (GAC) accounts for about 95% of gastric cancers. The levels of uPA and of the lncRNA TRPM2-AS (TRPM2-AS) are upregulated, whereas the level of miR-138-5p is downregulated in GAC samples compared with adjacent normal tissues. Indeed, miR-138-5p directly targets uPA mRNA. In GAC, TRPM2-AS recruits miR-138-5p, liberating uPA mRNA, which becomes available for translation. In fact, the effects of TRPM2-AS on cell proliferation, migration and apoptosis, impaired by TRPM2-AS silencing, can be partially restored by uPA overexpression [38].

In colorectal cancer (CRC) cell lines, the downregulation of miR-193a-3p induces the increase in its direct target uPA. Consistently, miR-193a-3p overexpression impairs CRC cell proliferation and migration, activities rescued by uPA overexpression [39]. 

In hepatocellular carcinoma (HCC) tissues, the levels of mature miR-193a are downregulated while uPA levels are generally higher as compared to their adjacent non-tumoral counterparts. The overexpression of miR-193a in HCC cells negatively regulates uPA and, at the same time, decreases their proliferation and increases apoptosis [40].

The uPA and the receptor tyrosine-kinase (RTK) c-met are usually overexpressed in HCC and are considered negative prognostic factors. Both molecules can be targeted by miR-23b, whose overexpression decreases their expression and impairs the migration ability of HCC cells. Accordingly, inhibition of the endogenous miR-23b by anti-miR-23b molecules leads to the upregulation of uPA and c-met expression in normal human fibroblasts [41].

miR-193b represses uPA and cyclin D expression and significantly reduces proliferation, migration and invasion in a non-small cell lung cancer (NSCLC) cell line, as compared to the control cells, while its inhibition increases the same activities in the same cells. Moreover, miR-193b is markedly downregulated in NSCLC tissues as compared to adjacent normal tissues [42]. 

In breast cancer cell lines, miR-193b directly targets uPA and significantly inhibits their invasion ability; in immunodeficient mouse models, miR-193b impairs the growth and dissemination of xenograft tumors probably by regulating the expression of uPA [43]. 

miR-193b also seems to be involved in the pro-tumoral effect of the cystic fibrosis (CF) transmembrane conductance regulator (CFTR). An association between cancer incidence and genetic variations in the CFTR gene has been suggested, even though the exact role of CFTR in cancer has not been elucidated. In prostate cancer (PC) cell lines and tissues, CFTR expression is significantly downregulated. Its overexpression in PC cells increases miRNA 193b expression, which, in turn, suppresses uPA production and impairs cell growth, adhesion and migration. Accordingly, CTFR silencing promotes a malignant phenotype both in vitro and in vivo, by increasing cell proliferation, invasion and migration; these activities can be reversed by overexpressing miR193b or by antibodies against uPA [44].

miR-645 directly targets uPA and decreases the invasive growth of triple-negative breast cancer (TNBC) cells both in vitro and in nude mice [45].

uPA and PAI-1 are involved in ECM deposition in hypertrophic scars (HS); miR-181c and miR-10a, differentially expressed in HS fibroblasts (HFs), target uPA and PAI-1, respectively, influencing HS pathogenesis [46].

Finally, Zhang and colleagues identified differentially expressed lncRNAs that play an important role in the pathogenesis of oral squamous cell carcinoma (OSCC); through a bioinformatics analysis, they found that the lncRNAs FTH1P3, PDIA3F, and GTF2IRD2P1 may be involved in OSCC progression and metastasis through the targeting of some key regulators of tumorigenesis, including uPA [47].

### 4.2. PAI-1 

PAI-1 is produced by various cell types, including platelets, hepatocytes and endothelial cells; its expression is regulated by several growth factors and cytokines. Increased plasma levels of PAI-1 have been observed in various tumors and represent a negative prognostic factor. Elevated plasma levels of PAI-1, in conjunction with the risk of atherosclerotic and atherothrombotic complications in some tumor types may worsen disease [48].

Several reports show that PAI-1 expression can be regulated by several miRNAs in tumors, influencing their development and progression.

The miR-143/-145 cluster is downregulated in all stages of bladder cancer and is inversely correlated with PAI-1 expression. Both mature miR-143 and miR-145 directly target the PAI-1 3′UTR, leading to reduced PAI-1 mRNA and protein levels [49].

The lncRNA MAFG-AS1, overexpressed in bladder cancer cell lines and tissues, contributes to tumorigenesis by acting as a molecular sponge for miR-143-3p, thus modulating PAI-1 levels [50].

miR-143 is also involved in the regulation of PAI-1 expression in human osteosarcoma cells. In fact, intravenous injection of miR-143 significantly suppresses the lung metastasis of human osteosarcoma cells in a mouse model, probably through the downregulation of PAI-1 expression which, in turn, is associated with reduced expression of the matrix metalloprotease 13 (MMP13) [51].

miR-30b, which promotes apoptosis and suppresses tumor growth by targeting PAI-1, is downregulated in gastric carcinoma (GC) cell lines and tissues [52].

miR-143-3p can be recruited by the lncRNA LINC00200, which is significantly overexpressed in GC tissues and cell lines. Indeed, LINC00200 acts as a molecular sponge for miR-143-3p, thus leading to the increase in its target PAI-1. The knockdown of LINC00200 in GC cells suppresses their proliferation, invasion and migration in vitro and inhibits tumorigenesis in mouse xenografts through a mechanism that probably involves PAI-1 [53]. 

A similar role has also been reported for the lncRNA NKX2-1-AS1, upregulated in GC cell lines and tissues. In fact, NKX2-1-AS1 recruits miR-145-5p, allowing the translation of its target PAI-1 and thus the activation of the VEGFR-2 signaling pathway, which promotes angiogenesis and tumor progression [54]. 

In colon adenocarcinoma cell lines, the upregulation of another lncRNA, LINC00491, has been observed. LINC00491 promotes proliferation, migration, and invasion of colon adenocarcinoma cells, probably by recruiting miR-145 which targets PAI-1, thus playing an oncogenic role during colon adenocarcinoma pathogenesis [55].

miR-486 impairs PAI-1 expression in human myxoid liposarcoma tissues [56].

A high PAI-1 level is a negative prognostic factor in NSCLC; indeed, PAI-1 is a target of miR-34a, whose level is reduced in NSCLC patients with metastasis. Increased PAI-1 promotes epithelial–mesenchymal transition (EMT) in NSCLC cells through the activation of the Stat3 signaling pathway, which transcriptionally suppresses miR-34a in a positive regulatory loop [57].

The sequencing of small RNAs obtained from biopsies at different stages along the malignant evolution of keratinocytes towards cutaneous squamous cell carcinoma (cSCC) showed variations in the levels of several miRs as compared to a normal epidermis. miR-497 was among the more underexpressed miRNAs. In fact, miR-497 targets PAI-1 and induces the reversion of the epithelial-to-mesenchymal transition. The reduced expression of miR-497 is associated with poor prognosis in human glioma, hepatocellular carcinoma, breast cancer, cervical cancer and renal cancer [58].

In pancreatic ductal adenocarcinoma (PDAC), characterized by very early metastasis, miR-192 expression is downregulated by promoter methylation; its overexpression reduces cell proliferation and invasion in vitro and in vivo by targeting PAI-1 and downregulating its expression [59].

TUC338 is a lncRNA containing elements that are fully conserved across human and rodent genomes and, for that, it is termed ultra-conserved lncRNA. TUC338 expression is upregulated in hepatocellular carcinoma cells and tissues and is involved in their growth. A direct physical interaction between TUC338 RNA and the PAI-1 RNA binding protein (PAI1-RBP) has been demonstrated, resulting in the positive posttranscriptional regulation of PAI-1 mRNA. Increased PAI-1 contributes to the oncogenic effects of enhanced TUC338 expression in HCC [60].

In glioblastoma (GBM) tissues, increased ZNF652 circRNA and PAI-1 and downregulation of miR-486 are associated with a poor prognosis. Indeed, ZNF652 upregulates PAI-1 expression in GBM cells by sponging miR-486, suggesting that targeting the ZNF652 circRNA may represent a novel and effective strategy to suppress cancer progression in GBM [61].

Finally, a reverse regulatory activity between lncRNAs and PAI-1 has also been observed in triple-negative breast cancer. High PAI-1 expression has a potential prognostic value in TNBC patients; in vitro, PAI-1 can induce the migration and invasion of TNBC cells. It has been shown that PAI-1 can promote the expression of the oncogenic lncRNA SOX2-OT which acts as a molecular sponge for the oncosuppressor miR-942-5p [62].

### 4.3. PAI-2

PAI-2 can be considered a stress protein since its expression is upregulated in activated macrophages and differentiating keratinocytes. PAI-2 transcription is stimulated by various inflammatory mediators and by viral and bacterial infections [63]. Its expression can be regulated at the transcriptional and posttranscriptional levels, through RBPs [63,64]. However, the miR-mediated regulation of PAI-2 has received little attention, probably because its role has not been fully elucidated and its involvement in cancer remains controversial [18]. 

In cholangiocarcinoma (CCA), the abundant tumor stroma plays a crucial role in cancer progression. A comparison between the miRNA expression profiles of CCA-associated fibroblasts (CCFs) and normal skin fibroblasts showed the downregulation of miR-15a in CCFs. PAI-2 has been identified as a target gene of miR-15a. The lower expression of miR-15a and higher expression of PAI-2 has been observed in human CCA samples compared with normal liver tissues and was associated with the increased migration of CCA cells [65].

miR-200c/141 indirectly upregulate PAI-2 by regulating PAI-2 transcription factors and miRNAs in breast cancer cells [20].

### 4.4. The uPAR

The uPAR protein is expressed at a low level in many different tissues; its expression significantly increases in various cancers and its upregulation represents a negative prognostic factor. The expression of uPAR can be regulated at the transcriptional and posttranscriptional levels. Posttranscriptional uPAR regulation involves stabilizing and destabilizing RBPs [66,67,68,69]. More recently, uPAR-targeting miRs have been identified. 

The co-regulation of the expression of uPAR and the chemokine receptor CXCR4 has been observed in acute myeloid leukemia (AML) cells and blasts; in fact, both receptors are directly targeted by the same miRs, i.e., miR-335, miR-146a and miR-622. The overexpression of all three of these miRs impairs the migration, invasion and proliferation of myelomonocytic cells [70]. The targeting of uPAR by miR-335 has also been confirmed in human periodontal ligament fibroblasts, in which miR-335 also targets the RANKL cytokine [71].

The uPAR protein is overexpressed and represents a negative prognostic marker in prostate carcinoma. In fact, uPAR is directly targeted by miR-143, which is downregulated and acts as a tumor suppressor in PC. The nanoparticle-mediated delivery of miR-143 reduces uPAR protein, but not its mRNA, thus indicating translational inhibition rather than mRNA degradation by this miR [72]. 

The expression of uPAR is indirectly modulated by miR-10b, which is overexpressed in glioma and directly associated with the glioma grade and malignancy. In fact, miR-10b directly targets HOXD10, thus leading to the upregulation of uPAR and MMP14 expression [73]. HOXD10 is also targeted by miR-378a; the overexpression of miR-378a enhances in vitro cell invasion and migration by indirectly inducing uPAR and MMP2 increase and promotes angiogenesis in vivo [74].

Finally, miR-221/-222 directly target the soluble form (isoform 2) of uPAR in triple-negative breast cancer cells. However, unlike other miRs, miR-221 seems to promote uPAR mRNA translation rather than suppressing it; consistently, the inhibition of miR-221 reduces the expression of uPAR protein and of vimentin and RHOC, two markers of tumor cell invasion. The authors of this study hypothesize that this positive regulation might be due to cofactors such as RBPs [75].

The miRNA-mediated regulation of uPAR expression leads to the inclusion of uPAR mRNA in the complex network connecting the different species of cellular RNAs. This aspect has been investigated by focusing on the ability of the 3′UTR of uPAR mRNA to influence the expression of other genes. Indeed, uPAR-3′UTR can positively regulate the expression of other mRNAs by recruiting common miRs, thus acting as a ceRNA. In fact, the transfection of an AML cell line with the uPAR 3′UTR, inserted downstream of a reporter gene, downregulated the reporter gene expression and increased endogenous uPAR expression. Transfection of the uPAR 3′UTR also increased the expression of CXCR4, which is regulated by the same miRs as uPAR, and of other pro-tumoral factors. Furthermore, transfected uPAR 3′UTR modulated cell adhesion and migration [76]. The hypothesis that uPAR variants containing the 3′UTR are expressed in malignant cells to enhance uPAR ceRNA activity has been proposed. Indeed, three variants of uPAR mRNA containing the 3′UTR have been identified in AML cell lines, two of which were also expressed in AML blasts, at higher levels as compared to CD34^+^ hematopoietic cells from healthy donors. The most abundant variant, lacking exon 5 (uPAR Δ5), has been cloned and transfected in AML KG1 cells. The presence of the 3′UTR conferred high instability to the uPAR Δ5 variant transcript, preventing its translation in protein; at the same time, its overexpression regulated the expression of some pro-tumoral factors previously reported to be regulated by the 3′UTR of uPAR, confirming its ceRNA activity, and increased transfected cell adhesion, migration and proliferation [77]. 

The miRs directly targeting PAS components are summarized in Table 1; lncRNA sponging miRs regulating the expression of PAS components are listed in Table 2.

**Figure 3:**
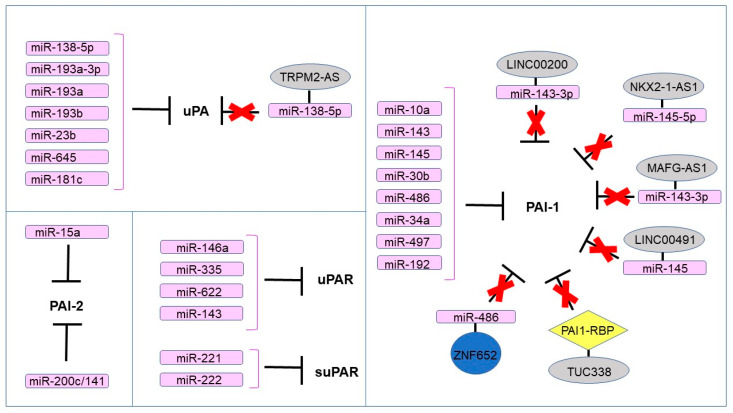
The ncRNAs regulating the expression of PAS components.

The effects of ncRNAs regulating the expression of PAS components are summarized in Figure 3.

## 5. PA-Targeting ncRNAs as Cancer Biomarkers

Some ncRNAs targeting PAS components are included in the list of ncRNAs proposed as plasma/EV biomarkers in various cancers [33]. Indeed, uPA-targeting miR-23b-3p, miR-138-5p and miR-193a are potential prognostic/diagnostic markers in hepatocellular, breast and colorectal carcinomas, respectively [78,79,80]. PAI-1-targeting miR-30b is predictive of the chemotherapy response in breast cancer [81], whereas PAI-1-targeting miR-486-5p has been proposed as a potential diagnostic biomarker in NSCLC [82] and miR-34a and miR-143 in osteosarcoma [83]. Finally, uPAR-targeting miR-146a is a potential diagnostic marker in breast cancer [84] and miR-222 in osteosarcoma [83].

Currently, licensed patents or clinical trials focused on the potential use of lncRNAs as diagnostic/prognostic biomarkers do not include lncRNAs involved in the regulation of the expression of PAS components [36].

## 6. Conclusions and Future Directions

The increased expression of uPA, PAI-1 and uPAR is a negative prognostic factor in most cancers. A large body of evidence has demonstrated the crucial role that each of them can play in tumor progression, which may or may not depend upon the regulation of proteolysis, through mechanisms sometimes not fully elucidated. However, the gradual acceptance that ncRNAs, once considered “junk” transcriptional products, are functional molecules regulating diverse biological processes [85] has opened new and unexpected scenarios. For instance, a mRNA, whose expression is negatively regulated by miRs, can in turn positively regulate the expression of other molecules by binding the same miRs. This view is further complicated by the observation that a transcript and its corresponding protein do not necessarily pursue the same objectives. In the context of cancer biology, for instance, the chemokine receptor CCR2, as protein, is implicated in cancer progression, but an increased CCR2 mRNA level is associated with the prolonged survival of breast cancer patients; in fact, CCR2 3′UTR impairs EMT in vitro and reduces breast cancer metastasis in vivo [86].

Further studies are required to fully elucidate the importance of ncRNAs in the PA system. It would be interesting, for instance, to investigate the role in cancer biology of PAI-1 mRNA, which is a target of various miRs, since, at protein level, it is a negative prognostic factor even though it inhibits the pro-tumoral proteolytic activity of uPA. Further, studies are required to assess the oncogenic role of uPAR mRNA in vivo. 

Finally, the potential roles that the PA system components might play in cancer biology at mRNA level, other than at protein level, should be investigated and taken into account in the search for novel anti-cancer therapeutic strategies. Currently, these are mainly focused only on the inhibition of the proteolytic or non-proteolytic functions of this complex system.

## Figures and Tables

**Table 1:** The miRs directly targeting PAS components. ↑: upregulated expression; ↓: downregulated expression; N.R.: not reported.

miRNA	Expression	Target	Cancer Type	Sample	Reference
miR-138-5p	↓	uPA	Gastric Adenocarcinoma	Tissues	[38]
miR-193a-3p	↓	uPA	Colorectal Cancer	Cell lines	[39]
miR-193a	↓	uPA	Hepatocellular Carcinoma	Tissues	[40]
miR-193b	↓ ↓	uPA	Non-Small Cell Lung Cancer Breast Cancer	Tissues Cell lines	[42] [43]
miR-23b	↓	uPA	Hepatocellular Carcinoma	Cell lines	[41]
miR-645	N.R.	uPA	Triple-negative Breast Cancer	Cell lines	[45]
miR-181c miR-10a	↑ ↓	uPA PAI-1	Hypertrophic Scars	Primary cell cultures Primary cell cultures	[46]
miR-143 miR-145	↓ ↓	PAI-1	Bladder Cancer	Tissues Tissues	[49]
miR-143	↓	PAI-1	Osteosarcoma	Tissues	[51]
miR-30b	↓	PAI-1	Gastric Adenocarcinoma	Cell lines; tissues	[52]
miR-486	↓	PAI-1	Myxoid Liposarcoma	Tissues	[56]
miR-34a	↓	PAI-1	Non-Small Cell Lung Cancer	Tissues	[57]
miR-497	↓	PAI-1	Cutaneous Squamous Cell Carcinoma	Tissues	[58]
miR-192	↓	PAI-1	Pancreatic Ductal Adenocarcinoma	Tissues	[59]
miR-15a	↓	PAI-2	Cholangiocarcinoma	Cell lines; tissues	[65]
miR-146a miR-335 miR-622	↓ ↓ ↓	uPAR	Acute Myeloid Leukemia	Cell lines; tissues Cell lines; tissues Cell lines	[70]
miR-143	↓	uPAR	Prostate Cancer	Tissues	[72]
miR-221/-222	↑	uPAR isoform 2	Triple-negative Breast Cancer	Cell lines	[75]

**Table 2:** The lncRNAs sponging miRs regulating the expression of PAS components. ↑: upregulated expression.

ncRNA	Expression	miRNAs	Target	Cancer Type	Sample	References
TRPM2-AS	↑	miR-138-5p	uPA	Gastric Adenocarcinoma	Cell lines; tissues	[38]
LINC00200	↑	miR-143-3p	PAI-1	Gastric Carcinoma	Cell lines; tissues	[53]
NKX2-1-AS1	↑	miR-145-5p	PAI-1	Gastric Carcinoma	Cell lines; tissues	[54]
MAFG-AS1	↑	miR-143-3p	PAI-1	Bladder Cancer	Cell lines; tissues	[50]
LINC00491	↑	miR-145	PAI-1	Colon Adenocarcinoma	Cell lines	[55]
circZNF652	↑	miR-486	PAI-1	Glioblastoma	Cell lines; tissues	[61]

## Data Availability

Not applicable.

## References

[1] DeVita V.T., Rosenberg S.A. (2012). Two Hundred Years of Cancer Research. N. Engl. J. Med..

[2] Shabalina S.A., Spiridonov N.A. (2004). The Mammalian Transcriptome and the Function of Non-Coding DNA Sequences. Genome Biol..

[3] Hombach S., Kretz M. (2016). Non-Coding RNAs: Classification, Biology and Functioning. Adv. Exp. Med. Biol..

[4] Slack F.J., Chinnaiyan A.M. (2019). The Role of Non-Coding RNAs in Oncology. Cell.

[5] Bharadwaj A.G., Holloway R.W., Miller V.A., Waisman D.M. (2021). Plasmin and Plasminogen System in the Tumor Microenvironment: Implications for Cancer Diagnosis, Prognosis, and Therapy. Cancers.

[6] Montuori N., Ragno P. (2009). Multiple Activities of a Multifaceted Receptor: Roles of Cleaved and Soluble UPAR. Front. Biosci..

[7] Rijken D.C., Lijnen H.R. (2009). New Insights into the Molecular Mechanisms of the Fibrinolytic System. J. Thromb. Haemost..

[8] Baker S.K., Strickland S. (2020). A Critical Role for Plasminogen in Inflammation. J. Exp. Med..

[9] Gebbink M.F.B.G. (2011). Tissue-Type Plasminogen Activator-Mediated Plasminogen Activation and Contact Activation, Implications in and beyond Haemostasis. J. Thromb. Haemost..

[10] Carmeliet P., Bouché A., De Clercq C., Janssen S., Pollefeyt S., Wyns S., Mulligan R.C., Collen D. (1995). Biological Effects of Disruption of the Tissue-Type Plasminogen Activator, Urokinase-Type Plasminogen Activator, and Plasminogen Activator Inhibitor-1 Genes in Mice. Ann. N. Y. Acad. Sci..

[11] Vassalli J.D., Baccino D., Belin D. (1985). A Cellular Binding Site for the Mr 55,000 Form of the Human Plasminogen Activator, Urokinase. J. Cell Biol..

[12] Carriero M.V., Franco P., Votta G., Longanesi-Cattani I., Vento M.T., Masucci M.T., Mancini A., Caputi M., Iaccarino I., Stoppelli M.P. (2011). Regulation of Cell Migration and Invasion by Specific Modules of UPA: Mechanistic Insights and Specific Inhibitors. Curr. Drug Targets.

[13] Mahmood N., Rabbani S.A. (2021). Fibrinolytic System and Cancer: Diagnostic and Therapeutic Applications. Int. J. Mol. Sci..

[14] Seiffert D., Loskutoff D.J. (1991). Evidence That Type 1 Plasminogen Activator Inhibitor Binds to the Somatomedin B Domain of Vitronectin. J. Biol. Chem..

[15] Chu Y., Bucci J.C., Peterson C.B. (2020). Identification of a PAI-1-Binding Site within an Intrinsically Disordered Region of Vitronectin. Protein Sci..

[16] Czekay R.-P., Wilkins-Port C.E., Higgins S.P., Freytag J., Overstreet J.M., Klein R.M., Higgins C.E., Samarakoon R., Higgins P.J. (2011). PAI-1: An Integrator of Cell Signaling and Migration. Int. J. Cell Biol..

[17] Ismail A.A., Shaker B.T., Bajou K. (2021). The Plasminogen-Activator Plasmin System in Physiological and Pathophysiological Angiogenesis. Int. J. Mol. Sci..

[18] Croucher D.R., Saunders D.N., Lobov S., Ranson M. (2008). Revisiting the Biological Roles of PAI2 (SERPINB2) in Cancer. Nat. Rev. Cancer.

[19] Westrick R.J., Røjkjaer L.P., Yang A.Y., Roh M.H., Siebert A.E., Ginsburg D. (2020). Deficiency of Plasminogen Activator Inhibitor-2 Results in Accelerated Tumor Growth. J. Thromb. Haemost..

[20] Jin T., Kim H.S., Choi S.K., Hwang E.H., Woo J., Ryu H.S., Kim K., Moon A., Moon W.K. (2017). MicroRNA-200c/141 Upregulates SerpinB2 to Promote Breast Cancer Cell Metastasis and Reduce Patient Survival. Oncotarget.

[21] Furuya H., Hayashi K., Shimizu Y., Kim N., Tsukikawa Y., Chen R., Sun Y., Chan O.T.M., Pagano I., Peres R. (2020). Plasminogen Activator Inhibitor-2 (PAI-2) Overexpression Supports Bladder Cancer Development in PAI-1 Knockout Mice in N-Butyl-N- (4-Hydroxybutyl)-Nitrosamine-Induced Bladder Cancer Mouse Model. J. Transl. Med..

[22] Roldan A.L., Cubellis M.V., Masucci M.T., Behrendt N., Lund L.R., Danø K., Appella E., Blasi F. (1990). Cloning and Expression of the Receptor for Human Urokinase Plasminogen Activator, a Central Molecule in Cell Surface, Plasmin Dependent Proteolysis. EMBO J..

[23] Gorrasi A., Petrone A.M., Li Santi A., Alfieri M., Montuori N., Ragno P. (2020). New Pieces in the Puzzle of UPAR Role in Cell Migration Mechanisms. Cells.

[24] Ellis V., Behrendt N., Danø K. (1991). Plasminogen Activation by Receptor-Bound Urokinase. A Kinetic Study with Both Cell-Associated and Isolated Receptor. J. Biol. Chem..

[25] Alfano D., Franco P., Stoppelli M.P. (2022). Modulation of Cellular Function by the Urokinase Receptor Signalling: A Mechanistic View. Front. Cell Dev. Biol..

[26] Li Santi A., Napolitano F., Montuori N., Ragno P. (2021). The Urokinase Receptor: A Multifunctional Receptor in Cancer Cell Biology. Therapeutic Implications. Int. J. Mol. Sci..

[27] Bartel D.P. (2018). Metazoan MicroRNAs. Cell.

[28] Peng Y., Croce C.M. (2016). The Role of MicroRNAs in Human Cancer. Signal Transduct. Target Ther..

[29] Tay Y., Rinn J., Pandolfi P.P. (2014). The Multilayered Complexity of CeRNA Crosstalk and Competition. Nature.

[30] Velázquez-Cruz A., Baños-Jaime B., Díaz-Quintana A., De la Rosa M.A., Díaz-Moreno I. (2021). Post-Translational Control of RNA-Binding Proteins and Disease-Related Dysregulation. Front. Mol. Biosci..

[31] Kopp F., Mendell J.T. (2018). Functional Classification and Experimental Dissection of Long Noncoding RNAs. Cell.

[32] Zhao X., Cai Y., Xu J. (2019). Circular RNAs: Biogenesis, Mechanism, and Function in Human Cancers. Int. J. Mol. Sci..

[33] Sarhadi V.K., Armengol G. (2022). Molecular Biomarkers in Cancer. Biomolecules.

[34] Lei B., Tian Z., Fan W., Ni B. (2019). Circular RNA: A Novel Biomarker and Therapeutic Target for Human Cancers. Int. J. Med. Sci..

[35] Qian Y., Shi L., Luo Z. (2020). Long Non-Coding RNAs in Cancer: Implications for Diagnosis, Prognosis, and Therapy. Front. Med..

[36] Aprile M., Costa V., Cimmino A., Calin G.A. (2022). Emerging Role of Oncogenic Long Noncoding RNA as Cancer Biomarkers. Int. J. Cancer.

[37] Menon A., Abd-Aziz N., Khalid K., Poh C.L., Naidu R. (2022). MiRNA: A Promising Therapeutic Target in Cancer. Int. J. Mol. Sci..

[38] Sun J., Zhou F., Xue J., Ji C., Qu Y., Pan Y. (2021). Long Non-Coding RNA TRPM2-AS Regulates MicroRNA MiR-138-5p and PLAU (Plasminogen Activator, Urokinase) to Promote the Progression of Gastric Adenocarcinoma. Bioengineered.

[39] Lin M., Zhang Z., Gao M., Yu H., Sheng H., Huang J. (2019). MicroRNA-193a-3p Suppresses the Colorectal Cancer Cell Proliferation and Progression through Downregulating the PLAU Expression. Cancer Manag. Res..

[40] Salvi A., Conde I., Abeni E., Arici B., Grossi I., Specchia C., Portolani N., Barlati S., De Petro G. (2013). Effects of MiR-193a and Sorafenib on Hepatocellular Carcinoma Cells. Mol. Cancer.

[41] Salvi A., Sabelli C., Moncini S., Venturin M., Arici B., Riva P., Portolani N., Giulini S.M., De Petro G., Barlati S. (2009). MicroRNA-23b Mediates Urokinase and c-Met Downmodulation and a Decreased Migration of Human Hepatocellular Carcinoma Cells. FEBS J..

[42] Hu H., Li S., Liu J., Ni B. (2012). MicroRNA-193b Modulates Proliferation, Migration, and Invasion of Non-Small Cell Lung Cancer Cells. Acta Biochim. Biophys Sin..

[43] Li X.-F., Yan P.-J., Shao Z.-M. (2009). Downregulation of MiR-193b Contributes to Enhance Urokinase-Type Plasminogen Activator (UPA) Expression and Tumor Progression and Invasion in Human Breast Cancer. Oncogene.

[44] Xie C., Jiang X.H., Zhang J.T., Sun T.T., Dong J.D., Sanders A.J., Diao R.Y., Wang Y., Fok K.L., Tsang L.L. (2013). CFTR Suppresses Tumor Progression through MiR-193b Targeting Urokinase Plasminogen Activator (UPA) in Prostate Cancer. Oncogene.

[45] Meng D., Lei M., Han Y., Zhao D., Zhang X., Yang Y., Liu R. (2018). MicroRNA-645 Targets Urokinase Plasminogen Activator and Decreases the Invasive Growth of MDA-MB-231 Triple-Negative Breast Cancer Cells. Onco Targets Ther..

[46] Li C., Zhu H.-Y., Bai W.-D., Su L.-L., Liu J.-Q., Cai W.-X., Zhao B., Gao J.-X., Han S.-C., Li J. (2015). MiR-10a and MiR-181c Regulate Collagen Type I Generation in Hypertrophic Scars by Targeting PAI-1 and UPA. FEBS Lett..

[47] Zhang S., Tian L., Ma P., Sun Q., Zhang K., Wang G., Liu H., Xu B. (2015). Potential Role of Differentially Expressed LncRNAs in the Pathogenesis of Oral Squamous Cell Carcinoma. Arch. Oral. Biol..

[48] Li S., Wei X., He J., Tian X., Yuan S., Sun L. (2018). Plasminogen Activator Inhibitor-1 in Cancer Research. Biomed. Pharmacother..

[49] Villadsen S.B., Bramsen J.B., Ostenfeld M.S., Wiklund E.D., Fristrup N., Gao S., Hansen T.B., Jensen T.I., Borre M., Ørntoft T.F. (2012). The MiR-143/-145 Cluster Regulates Plasminogen Activator Inhibitor-1 in Bladder Cancer. Br. J. Cancer.

[50] Sun X., Cai Y., Hu X., Mo M., Zhao C., He W., Li Y. (2020). Long Noncoding RNA MAFG-AS1 Facilitates Bladder Cancer Tumorigenesis via Regulation of MiR-143-3p/SERPINE1 Axis. Transl. Cancer Res..

[51] Hirahata M., Osaki M., Kanda Y., Sugimoto Y., Yoshioka Y., Kosaka N., Takeshita F., Fujiwara T., Kawai A., Ito H. (2016). PAI-1, a Target Gene of MiR-143, Regulates Invasion and Metastasis by Upregulating MMP-13 Expression of Human Osteosarcoma. Cancer Med..

[52] Zhu E.-D., Li N., Li B.-S., Li W., Zhang W.-J., Mao X.-H., Guo G., Zou Q.-M., Xiao B. (2014). MiR-30b, Down-Regulated in Gastric Cancer, Promotes Apoptosis and Suppresses Tumor Growth by Targeting Plasminogen Activator Inhibitor-1. PLoS ONE.

[53] He W., Zhang D., Li D., Zhu D., Geng Y., Wang Q., He J., Wu J. (2021). Knockdown of Long Non-Coding RNA LINC00200 Inhibits Gastric Cancer Progression by Regulating MiR-143-3p/SERPINE1 Axis. Dig. Dis. Sci..

[54] Teng F., Zhang J.-X., Chen Y., Shen X.-D., Su C., Guo Y.-J., Wang P.-H., Shi C.-C., Lei M., Cao Y.-O. (2021). LncRNA NKX2-1-AS1 Promotes Tumor Progression and Angiogenesis via Upregulation of SERPINE1 Expression and Activation of the VEGFR-2 Signaling Pathway in Gastric Cancer. Mol. Oncol..

[55] Wan J., Deng D., Wang X., Wang X., Jiang S., Cui R. (2019). LINC00491 as a New Molecular Marker Can Promote the Proliferation, Migration and Invasion of Colon Adenocarcinoma Cells. Onco Targets Ther..

[56] Borjigin N., Ohno S., Wu W., Tanaka M., Suzuki R., Fujita K., Takanashi M., Oikawa K., Goto T., Motoi T. (2012). TLS-CHOP Represses MiR-486 Expression, Inducing Upregulation of a Metastasis Regulator PAI-1 in Human Myxoid Liposarcoma. Biochem. Biophys Res. Commun..

[57] Lin X., Lin B., Chen X., Zhang B., Xiao X., Shi J., Lin J., Chen X. (2017). PAI-1/PIAS3/Stat3/MiR-34a Forms a Positive Feedback Loop to Promote EMT-Mediated Metastasis through Stat3 Signaling in Non-Small Cell Lung Cancer. Biochem Biophys Res. Commun..

[58] Mizrahi A., Barzilai A., Gur-Wahnon D., Ben-Dov I.Z., Glassberg S., Meningher T., Elharar E., Masalha M., Jacob-Hirsch J., Tabibian-Keissar H. (2018). Alterations of MicroRNAs throughout the Malignant Evolution of Cutaneous Squamous Cell Carcinoma: The Role of MiR-497 in Epithelial to Mesenchymal Transition of Keratinocytes. Oncogene.

[59] Botla S.K., Savant S., Jandaghi P., Bauer A.S., Mücke O., Moskalev E.A., Neoptolemos J.P., Costello E., Greenhalf W., Scarpa A. (2016). Early Epigenetic Downregulation of MicroRNA-192 Expression Promotes Pancreatic Cancer Progression. Cancer Res..

[60] Wen H.-J., Walsh M.P., Yan I.K., Takahashi K., Fields A., Patel T. (2018). Functional Modulation of Gene Expression by Ultraconserved Long Non-Coding RNA TUC338 during Growth of Human Hepatocellular Carcinoma. iScience.

[61] Liu L., Xiao S., Wang Y., Zhu Z., Cao Y., Yang S., Mai R., Zheng Y. (2022). Identification of a Novel Circular RNA CircZNF652/MiR-486-5p/SERPINE1 Signaling Cascade That Regulates Cancer Aggressiveness in Glioblastoma (GBM). Bioengineered.

[62] Zhang W., Yang S., Chen D., Yuwen D., Zhang J., Wei X., Han X., Guan X. (2022). SOX2-OT Induced by PAI-1 Promotes Triple-Negative Breast Cancer Cells Metastasis by Sponging MiR-942-5p and Activating PI3K/Akt Signaling. Cell Mol. Life Sci..

[63] Lee J.A., Cochran B.J., Lobov S., Ranson M. (2011). Forty Years Later and the Role of Plasminogen Activator Inhibitor Type 2/SERPINB2 Is Still an Enigma. Semin. Thromb. Hemost.

[64] Tierney M.J., Medcalf R.L. (2001). Plasminogen Activator Inhibitor Type 2 Contains mRNA Instability Elements within Exon 4 of the Coding Region: Sequence Homology to Coding Region Instability Determinants in Other mRNAs. J. Biol. Chem..

[65] Utaijaratrasmi P., Vaeteewoottacharn K., Tsunematsu T., Jamjantra P., Wongkham S., Pairojkul C., Khuntikeo N., Ishimaru N., Sirivatanauksorn Y., Pongpaibul A. (2018). The MicroRNA-15a-PAI-2 Axis in Cholangiocarcinoma-Associated Fibroblasts Promotes Migration of Cancer Cells. Mol. Cancer.

[66] Montuori N., Mattiello A., Mancini A., Santoli M., Taglialatela P., Caputi M., Rossi G., Ragno P. (2001). Urokinase-Type Plasminogen Activator up-Regulates the Expression of Its Cellular Receptor through a Post-Transcriptional Mechanism. FEBS Lett..

[67] Montuori N., Rossi G., Ragno P. (2002). Post-Transcriptional Regulation of Gene Expression in the Plasminogen Activation System. Biol. Chem..

[68] Montuori N., Mattiello A., Mancini A., Taglialatela P., Caputi M., Rossi G., Ragno P. (2003). Urokinase-Mediated Posttranscriptional Regulation of Urokinase-Receptor Expression in Non Small Cell Lung Carcinoma. Int. J. Cancer.

[69] Nagamine Y., Medcalf R.L., Muñoz-Cánoves P. (2005). Transcriptional and Posttranscriptional Regulation of the Plasminogen Activator System. Thromb. Haemost..

[70] Alfano D., Gorrasi A., Li Santi A., Ricci P., Montuori N., Selleri C., Ragno P. (2015). Urokinase Receptor and CXCR4 Are Regulated by Common MicroRNAs in Leukaemia Cells. J. Cell. Mol. Med..

[71] Yue J., Wang P., Hong Q., Liao Q., Yan L., Xu W., Chen X., Zheng Q., Zhang L., Huang D. (2017). MicroRNA-335-5p Plays Dual Roles in Periapical Lesions by Complex Regulation Pathways. J. Endod..

[72] Wach S., Brandl M., Borchardt H., Weigelt K., Lukat S., Nolte E., Al-Janabi O., Hart M., Grässer F., Giedl J. (2019). Exploring the MIR143-UPAR Axis for the Inhibition of Human Prostate Cancer Cells In Vitro and In Vivo. Mol. Nucleic Acids.

[73] Sun L., Yan W., Wang Y., Sun G., Luo H., Zhang J., Wang X., You Y., Yang Z., Liu N. (2011). MicroRNA-10b Induces Glioma Cell Invasion by Modulating MMP-14 and UPAR Expression via HOXD10. Brain Res..

[74] Tupone M.G., D’Aguanno S., Di Martile M., Valentini E., Desideri M., Trisciuoglio D., Donzelli S., Sacconi A., Buglioni S., Ercolani C. (2020). MicroRNA-378a-5p Is a Novel Positive Regulator of Melanoma Progression. Oncogenesis.

[75] Falkenberg N., Anastasov N., Schaub A., Radulovic V., Schmitt M., Magdolen V., Aubele M. (2015). Secreted UPAR Isoform 2 (UPAR7b) Is a Novel Direct Target of MiR-221. Oncotarget.

[76] Li Santi A., Gorrasi A., Alfieri M., Montuori N., Ragno P. (2018). A Novel Oncogenic Role for Urokinase Receptor in Leukemia Cells: Molecular Sponge for Oncosuppressor MicroRNAs. Oncotarget.

[77] Alfieri M., Li Santi A., Meo L., Giudice V., Selleri C., Ragno P. (2022). Identification of UPAR Variants Acting as CeRNAs in Leukaemia Cells. Cancers.

[78] Manganelli M., Grossi I., Ferracin M., Guerriero P., Negrini M., Ghidini M., Senti C., Ratti M., Pizzo C., Passalacqua R. (2021). Longitudinal Circulating Levels of MiR-23b-3p, MiR-126-3p and LncRNA GAS5 in HCC Patients Treated with Sorafenib. Biomedicines.

[79] Xun J., Du L., Gao R., Shen L., Wang D., Kang L., Chen C., Zhang Z., Zhang Y., Yue S. (2021). Cancer-Derived Exosomal MiR-138-5p Modulates Polarization of Tumor-Associated Macrophages through Inhibition of KDM6B. Theranostics.

[80] Cho W.-C., Kim M., Park J.W., Jeong S.-Y., Ku J.-L. (2021). Exosomal MiR-193a and Let-7g Accelerate Cancer Progression on Primary Colorectal Cancer and Paired Peritoneal Metastatic Cancer. Transl. Oncol..

[81] Todorova V.K., Byrum S.D., Gies A.J., Haynie C., Smith H., Reyna N.S., Makhoul I. (2022). Circulating Exosomal MicroRNAs as Predictive Biomarkers of Neoadjuvant Chemotherapy Response in Breast Cancer. Curr. Oncol..

[82] Wu Q., Yu L., Lin X., Zheng Q., Zhang S., Chen D., Pan X., Huang Y. (2020). Combination of Serum MiRNAs with Serum Exosomal MiRNAs in Early Diagnosis for Non-Small-Cell Lung Cancer. Cancer Manag. Res..

[83] Gao S.-S., Wang Y.-J., Zhang G.-X., Zhang W.-T. (2020). Potential Diagnostic Value of MiRNAs in Peripheral Blood for Osteosarcoma: A Meta-Analysis. J. Bone Oncol..

[84] Li S., Zhang M., Xu F., Wang Y., Leng D. (2021). Detection Significance of MiR-3662, MiR-146a, and MiR-1290 in Serum Exosomes of Breast Cancer Patients. J. Cancer Res..

[85] Anastasiadou E., Jacob L.S., Slack F.J. (2018). Non-Coding RNA Networks in Cancer. Nat. Rev. Cancer.

[86] Hu J., Li X., Guo X., Guo Q., Xiang C., Zhang Z., Xing Y., Xi T., Zheng L. (2017). The CCR2 3′UTR Functions as a Competing Endogenous RNA to Inhibit Breast Cancer Metastasis. J. Cell Sci..

